# Rapid Induction of Aldosterone Synthesis in Cultured Neonatal Rat Cardiomyocytes under High Glucose Conditions

**DOI:** 10.1155/2013/161396

**Published:** 2013-10-28

**Authors:** Masami Fujisaki, Tomohisa Nagoshi, Tetsuo Nishikawa, Taro Date, Michihiro Yoshimura

**Affiliations:** ^1^Division of Cardiology, Department of Internal Medicine, The Jikei University School of Medicine, 3-25-8, Nishi-Shinbashi, Minato-Ku, Tokyo 105-8461, Japan; ^2^Endocrinology and Diabetes Center, Yokohama Rosai Hospital, 3211 Kozukue-Cho, Kohoku-Ku, Yokohama City, Kanagawa, 222-0036, Japan

## Abstract

In addition to classical adrenal cortical biosynthetic pathway, there is increasing evidence that aldosterone is produced in extra-adrenal tissues. Although we previously reported aldosterone production in the heart, the concept of cardiac aldosterone synthesis remains controversial. This is partly due to lack of established experimental models representing aldosterone synthase (CYP11B2) expression in robustly reproducible fashion. We herein investigated suitable conditions in neonatal rat cardiomyocytes (NRCMs) culture system producing CYP11B2 with considerable efficacy. NRCMs were cultured with various glucose doses for 2–24 hours. CYP11B2 mRNA expression and aldosterone concentrations secreted from NRCMs were determined using real-time PCR and enzyme immunoassay, respectively. We found that suitable conditions for CYP11B2 induction included four-hour incubation with high glucose conditions. Under these particular conditions, CYP11B2 expression, in accordance with aldosterone secretion, was significantly increased compared to those observed in the cells cultured under standard-glucose condition. Angiotensin II receptor blocker partially inhibited this CYP11B2 induction, suggesting that there is local renin-angiotensin-aldosterone system activation under high glucose conditions. The suitable conditions for CYP11B2 induction in NRCMs culture system are now clarified: high-glucose conditions with relatively brief period of culture promote CYP11B2 expression in cardiomyocytes. The current system will help to accelerate further progress in research on cardiac tissue aldosterone synthesis.

## 1. Introduction

Aldosterone, the last component of the renin-angiotensin-aldosterone system (RAAS), plays a pivotal role in the pathophysiology of cardiovascular diseases primarily through mineralocorticoid-receptor- (MR-) dependent actions [1]. Large clinical trials of patients with heart failure have shown that MR antagonists improve morbidity and mortality in conjunction with the current standard of care [2–4]. However, the circulating plasma aldosterone levels were within the physiological range in these large trials, thus suggesting the critical role of local cardiac RAAS activation in the pathogenesis of heart failure. 

There is increasing evidence that aldosterone, previously thought to be synthesized solely in the adrenal cortex, is also produced in extra-adrenal tissues, such as the heart, particularly in pathological states [5–13]. We have also previously reported that aldosterone is produced and the mRNA expression of aldosterone synthase (CYP11B2), the enzyme that catalyzes the terminal or key step in the synthesis of aldosterone, is induced in failing or hypertensive human ventricles [14–17], as well as in cultured neonatal rat cardiomyocytes (NRCMs) [18]. On the other hand, a few reports have demonstrated that aldosterone is unlikely to be synthesized in the heart in some experimental models [19–22]. Therefore, aldosterone synthesis in cardiomyocytes remains controversial. Alternatively, it is also possible that the degree of aldosterone synthesis varies delicately with culture conditions, as well as with the experimental models used [7]. Whatever the case, thus far, there are no established suitable experimental models clearly showing cardiac aldosterone production. 

High glucose conditions have been shown to induce the upregulation as well as activation of the local renin-angiotensin system in both *in vitro* and *in vivo* models of tissues including the heart [23–27]. Moreover, it has also been reported that the expression of CYP11B2 is increased under high glucose conditions in the kidneys [28, 29]. Therefore, we herein hypothesized that high glucose induces the upregulation of CYP11B2 and promotes aldosterone synthesis in cardiomyocytes. The aim of this study was to investigate the effects of high glucose conditions on aldosterone synthesis in cardiomyocytes and to explore suitable conditions for a NRCMs culture system inducing the upregulation of CYP11B2 in a robustly reproducible fashion. 

## 2. Materials and Methods

### 2.1. Primary Cultures of Isolated Neonatal Rat Cardiomyocytes (NRCMs)

All animal procedures conformed to the National Institutes of Health Guide for the Care and Use of Laboratory Animals and were approved by the Animal Research Committee at Jikei University School of Medicine. Primary myocyte cultures were prepared from 1- to 3-day-old Sprague-Dawley rat heart ventricles using the neonatal cardiomyocyte isolation system (Worthington Biochemical Corp), plated in 60 mm collagen-coated dishes as previously described [30, 31]. Cardiomyocytes were cultured in Dulbecco's modified eagle medium (DMEM) containing 5.5 mM of glucose/10% horse serum/5% fetal bovine serum for 48 hours with bromodeoxyuridine (200 *μ*mol/L) to inhibit the proliferation of nonmyocytes. The medium was then changed to serum-free DMEM with 5.5 mM glucose, and the cells were incubated for 18 hours before all experiments were performed in order to avoid the possible contamination with aldosterone or other neurohumoral factors contained in the serum. The culture conditions at this point were defined as “baseline” in the present study.

Following serum starvation, the NRCMs were exposed to normal glucose (5.5 mM) and escalating doses of high glucose (15, 25, and 40 mM) or mannitol-containing media to adjust the medium to an equivalent osmolarity (i.e., 336.3 mOsm/kgH_2_O in high glucose (25 mM) and the matched control with a normal glucose level (5.5 mM) with 19.5 mM mannitol) for the indicated periods (two to 24 hours). To examine the effects of angiotensin II receptor blocker (ARB), NRCMs were treated with or without 10^−7^ M of losartan (Sigma-Aldrich) for four hours. Next, the cells were frozen for RNA extraction. 

### 2.2. Isolation of Total RNA, Reverse Transcription (RT), and Real-Time Polymerase Chain Reaction (PCR)

 Total RNA was extracted from the frozen cells using TRIzol reagent (Invitrogen) and cleaned using a PureLink RNA Mini kit with PureLink DNase treatment (Invitrogen) according to the manufacturer's protocol. RNA was reverse transcribed using a high capacity reverse transcription kit (Applied Biosystems). RT was carried out for one cycle at 25°C for 10 minutes, 37°C for 120 minutes, and 85°C for five seconds. To evaluate the expression level of CYP11B2 mRNA, quantitative real-time PCR (qPCR) was performed using the StepOnePlus Real-time PCR System and the StepOne Software program (Applied Biosystems). The real-time PCR protocol consisted of one cycle at 95°C for 20 seconds followed by 50 cycles at 95°C for one second and 60°C for 20 seconds using the primers for CYP11B2 (Applied Biosystems, Rn01767818_g1) and 18S ribosomal RNA (Applied Biosystems, 4319413E). The transcriptional levels were determined using the ΔΔCt method with normalization to 18S. 

### 2.3. Measurement of the Aldosterone Levels

 The concentration of aldosterone in the conditioned media was measured with the Aldosterone Express EIA kit (Cayman Chemical) as previously described [32] after being concentrated with dichloromethane (Wako). The aldosterone levels were normalized to the total cellular protein amount per dish, which was determined according to the Bradford method (Bio-Rad).

### 2.4. Statistical Analysis

The data are presented as the mean ± SEM of at least four independent experiments. The statistical analyses were performed using one-way ANOVA followed by appropriate post hoc tests for multiple comparison correction and either Student's *t*-test or Wilcoxon's rank sum test for two data sets. *P* < 0.05 was considered to be significant.

## 3. Results

### 3.1. High Glucose Promotes the CYP11B2 mRNA Expression in NRCMs

**Figure 1:**
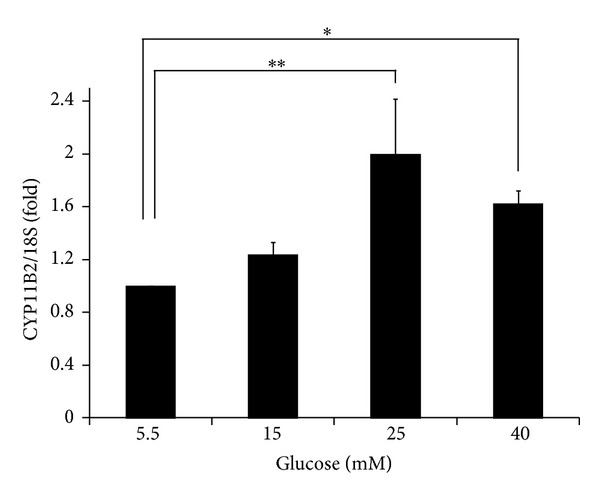
Quantification of the CYP11B2 transcript levels in NRCMs after four hours of incubation with the indicated glucose concentrations (5.5 mM, *n* = 15; 15 mM, *n* = 4; 25 mM, *n* = 15; 40 mM, *n* = 4). The qPCR data were normalized to 18S rRNA. The data are shown as the fold changes normalized to the levels found in NRCMs incubated with 5.5 mM glucose. **P < 0.01 and *P < 0.03 versus 5.5 mM glucose.

After four hours of incubation under high glucose conditions (25 and 40 mM), the expression levels of CYP11B2 mRNA in the NRCMs were significantly increased compared to those observed in the cells incubated under normal glucose conditions (5.5 mM) (2.00 ± 0.42-fold and 1.62 ± 0.10-fold, resp., Figure 1). These data suggest that high glucose conditions induce local aldosterone production in cardiomyocytes.

### 3.2. Time Course of the CYP11B2 Expression Stimulated by High Glucose

**Figure 2:**
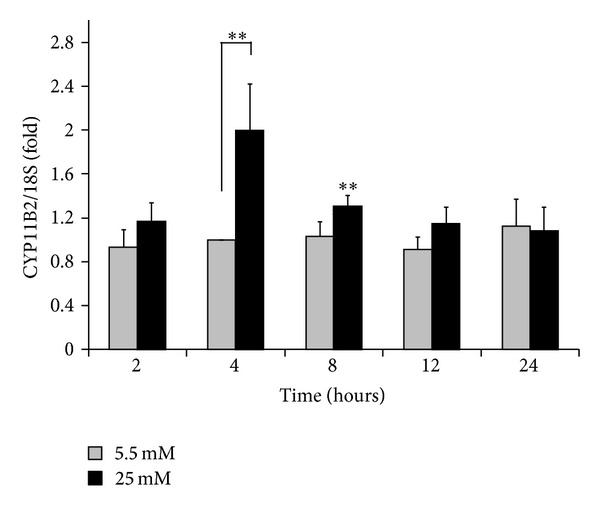
Time course of the relative expression of CYP11B2 mRNA in NRCMs. NRCMs were grown in culture medium containing either 5.5 mM (gray) or 25 mM (black) glucose for the indicated periods of time (2 hours, *n* = 6 each; 4 hours, *n* = 15 each; 8 hours, *n* = 5 each; 12 hours, *n* = 6 each; 24 hours, *n* = 5 each). The qPCR data were normalized to 18S rRNA. The results obtained from cells cultured under normal glucose conditions (gray bars) are shown as the fold changes normalized to the levels after 5.5 mM exposure for four hours, whereas the significance of the differences in the high glucose concentration (black bars) was assessed by comparison of the data with those from the corresponding normal glucose controls (**P < 0.01).

We next examined the time course of the effects of high glucose conditions (25 mM) on CYP11B2 induction in the NRCMs. Normal glucose conditions (5.5 mM) did not significantly alter the expression level of CYP11B2 mRNA at any time during incubation (Figure 2). Incubation in high glucose (25 mM) media significantly increased the CYP11B2 level, which showed a maximal effect at four hours, and to a lesser extent at eight hours, compared to that of the corresponding normal glucose controls (*P* < 0.01), while there were no significant differences at the other time points. Taken together, these data suggest a relatively rapid response of CYP11B2 induction to high glucose incubation in NRCMs. 

### 3.3. High Glucose Promotes Aldosterone Production and Secretion in NRCMs

**Figure 3:**
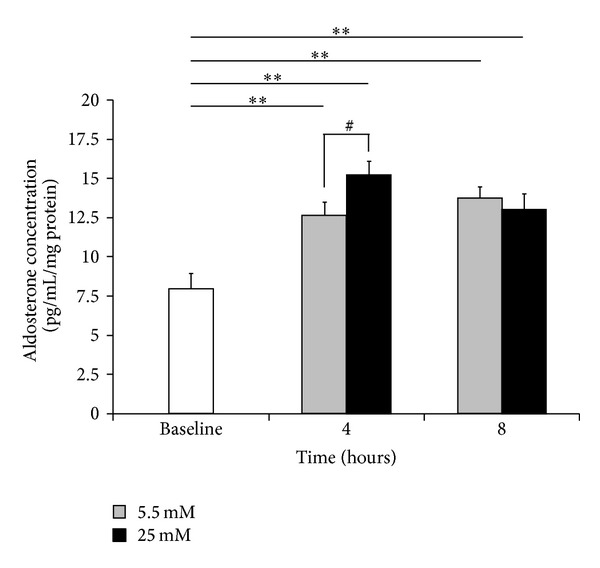
Time course of high glucose-stimulated aldosterone secretion from cardiomyocytes into the culture medium. NRCMs were grown in culture medium containing either 5.5 mM (gray) or 25 mM (black) glucose for the indicated periods of time (4 and 8 hours, *n* = 14 each). The white bar indicates the aldosterone concentration in the culture medium at baseline after primary myocyte culture, which was followed by an 18-hour serum-free incubation, as described in Section 2 (*n* = 12). The aldosterone concentration secreted into the culture medium was measured using an enzyme immunoassay. **P < 0.01 versus baseline, ^#^ *P* < 0.05 versus 5.5 mM glucose at four hours.

Intriguingly, the aldosterone concentration in the culture medium was increased even under normal glucose conditions (5.5 mM) in serum-free medium (12.66 ± 0.86 pg/mL/mg protein at four hours and 13.76 ± 0.72 pg/mL/mg protein at eight hours, resp., Figure 3). The aldosterone concentration was significantly increased after four hours of incubation under high glucose conditions (25 mM) compared to that observed in the cells incubated under normal glucose conditions (5.5 mM) for the same time period (15.23 ± 0.87 pg/mL/mg protein under high glucose conditions, *P* < 0.05). These data indicate that aldosterone is secreted from NRCMs, at least under serum-free conditions, and that high glucose conditions accelerate aldosterone production/secretion in accordance with CYP11B2 mRNA induction. 

### 3.4. The Inhibitory Effects of ARB on the High Glucose-Induced CYP11B2 Expression in NRCMs

**Figure 4:**
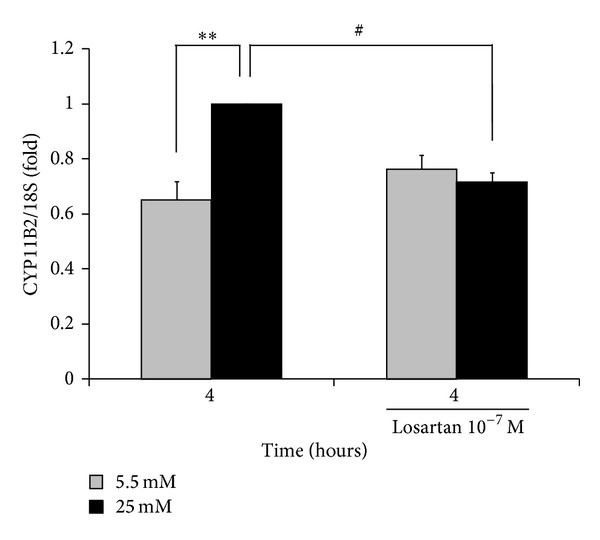
The inhibitory effects of ARB, losartan, on the high glucose-induced CYP11B2 expression in NRCMs. NRCMs were grown in culture medium containing either 5.5 mM (gray) or 25 mM (black) glucose for four hours, with or without 10^−7^ M of losartan (*n* = 11 each). The qPCR data were normalized to 18S rRNA expression. The data are shown as the fold changes normalized to the levels found in NRCMs incubated with 25 mM glucose alone for four hours. **P < 0.01 and ^#^ *P* < 0.02 versus 25 mM glucose at four hours.

To evaluate the role of the local endogenous angiotensin II cascade in this high glucose-induced CYP11B2 expression in NRCMs, we examined the effects of losartan (an ARB which blocks the intracellular angiotensin II receptors, rather than angiotensin II receptor internalization from the cell surface) on the CYP11B2 expression stimulated by high glucose conditions (25 mM). Losartan significantly, but not completely, reduced the CYP11B2 induction at four hours compared to the corresponding high glucose controls (28% reduction, *P* < 0.02, Figure 4).

## 4. Discussion

In this study, we demonstrated that high glucose conditions over a relatively short period promote the expression of aldosterone synthase (CYP11B2) and the production/secretion of aldosterone in NRCMs. We found that both the glucose concentration in the medium and the time course are critical factors for the conditions of the NRCMs culture system to detect the upregulation of CYP11B2 in a robustly reproducible fashion.

 The precise mechanisms of CYP11B2 induction under high glucose conditions are unknown. The regulation of aldosterone biosynthesis is divided into two phases: acute regulation via steroidogenic acute regulatory (StAR) proteins and chronic regulation via CYP11B2 [33–35]. Aldosterone is classically produced by the zona glomerulosa in the adrenal cortices, and its production is largely dependent on the expression level of the CYP11B2 gene, which is regulated by various factors, including the renin-angiotensin system [1, 33]. Regarding extra-adrenal aldosterone synthesis, it has been reported that, in the renal cortex, including cultured podocytes, high glucose levels increases the local renin, and angiotensin II levels, leading to the upregulation of the CYP11B2 mRNA expression [24, 28, 29]. Similarly, high glucose levels have been shown to increase the intracellular renin, angiotensinogen and angiotensin II levels in cardiomyocytes [25] and cardiac fibroblasts [26] as well as both murine [27] and human [23] diabetic heart tissues. Therefore, we speculate that the increased CYP11B2 expression observed in high glucose-stimulated NRCMs is partly attributed to the increased angiotensin II levels under high glucose conditions. In fact, we found that blocking both exogenous and endogenous angiotensin II cascades with losartan significantly reduced the high glucose-induced intracellular CYP11B2 expression in the present study (Figure 4). On the other hand, increased aldosterone secretion in the culture medium was also observed under normal glucose conditions (Figure 3), even though CYP11B2 mRNA expression level was not significantly altered (Figure 2). The aldosterone secretion from cardiomyocytes would be slightly induced by serum starvation *per se* (which causes considerable stress to the cardiomyocytes, leading to the activation of miscellaneous stress-related signaling cascades, as well as inflammatory reactions, such as reactive oxygen species production) even in the presence of low but continuous expression of CYP11B2 mRNA for two to eight hours. Other aldosterone biosynthetic mechanisms, such as StAR, or the regulation of aldosterone secretion from cardiomyocytes may also be involved [32, 36], but further studies are required to elucidate the precise mechanism. 

 The findings of the present study in some ways argue against previous negative data for cardiac aldosterone synthesis [19–22]. This discrepancy may be partly explained by differences in the models used, especially with respect to the culture conditions, species and time course of stimulation [7]. We previously reported that low-density lipoprotein as well as glucose induces local aldosterone production in renal mesangial cells starting at a relatively early period (four to six hours) [37, 38]. In the current study, we found that the time course is critical for high glucose-induced local aldosterone synthesis in cardiomyocytes: rapid induction of aldosterone synthesis for four hours of high glucose stimulation. 

There may be a pathophysiological role of the rapid induction of cardiac aldosterone synthesis (even a small amount) in response to high glucose conditions as a compensatory mechanism. Glucose, rather than fatty acids, becomes an important preferential substrate for metabolism and ATP generation under specific pathological conditions, such as ischemia and other types of heart failure [39]. Aldosterone, via its MR-independent actions, may transiently optimize the use of this limited alternative energy source via the rapid activation of Akt signaling, as we recently reported [31], although the persistent activity of aldosterone, via its MR-dependent actions, induces insulin resistance, which prevents the adaptive metabolic shift from fatty acid utilization toward glucose utilization [39]. Moreover, we recently reported that there is a transient decrease in serum potassium level in response to hyperglycemia during ischemic attack of acute coronary syndrome (ACS), which would be at least partially mediated through the aldosterone cascades [40].

## 5. Conclusions

We herein demonstrated that CYP11B2 gene is present in cultured NRCMs and reported for the first time that the expression of CYP11B2 as well as aldosterone secretion is promoted under high glucose conditions over a relatively brief period. The present system will help to contribute to achieving further progress in research of tissue aldosterone synthesis, particularly in the heart, and elucidate the critical role of local aldosterone in the pathophysiology of cardiovascular diseases, including ACS and heart failure.
